# A public health approach for deciding policy on infant feeding and mother–infant contact in the context of COVID-19

**DOI:** 10.1016/S2214-109X(20)30538-6

**Published:** 2021-02-22

**Authors:** Nigel Rollins, Nicole Minckas, Fyezah Jehan, Rakesh Lodha, Daniel Raiten, Claire Thorne, Philippe Van de Perre, Mija Ververs, Neff Walker, Rajiv Bahl, Cesar G Victora, WHO COVID-19 Maternal, Newborn, Child and Adolescent Health Research Network, Newborn and Infant Feeding Working Groups

**Affiliations:** a Department of Maternal, Newborn, Child and Adolescent Health and Ageing, World Health Organization, Geneva, Switzerland; b Department of Pediatrics and Child Health, Aga Khan University, Karachi, Pakistan; c Department of Pediatrics, All India Institute of Medical Sciences, New Delhi, India; d Eunice Kennedy Shriver National Institute of Child Health and Human Development, National Institutes of Health, Bethesda, MD, USA; e UCL Great Ormond Street Institute of Child Health, University College London, London, UK; f Pathogenesis and Control of Chronic Infections, INSERM, Etablissement Français du Sang, University of Montpellier, CHU Montpellier, Montpellier, France; g Johns Hopkins Bloomberg School of Public Health, Baltimore, MD, USA; h International Center for Equity in Health, Federal University of Pelotas, Pelotas, Brazil

## Abstract

The COVID-19 pandemic has raised concern about the possibility and effects of mother–infant transmission of SARS-CoV-2 through breastfeeding and close contact. The insufficient available evidence has resulted in differing recommendations by health professional associations and national health authorities. We present an approach for deciding public health policy on infant feeding and mother–infant contact in the context of COVID-19, or for future emerging viruses, that balances the risks that are associated with viral infection against child survival, lifelong health, and development, and also maternal health. Using the Lives Saved Tool, we used available data to show how different public health approaches might affect infant mortality. Based on existing evidence, including population and survival estimates, the number of infant deaths in low-income and middle-income countries due to COVID-19 (2020–21) might range between 1800 and 2800. By contrast, if mothers with confirmed SARS-CoV-2 infection are recommended to separate from their newborn babies and avoid or stop breastfeeding, additional deaths among infants would range between 188 000 and 273 000.

## Introduction

Exclusive and continued breastfeeding, skin-to-skin contact initiated in the first hour of birth, and responsive caregiving are strongly recommended by WHO for all infants and young children. Kangaroo mother care is als strongly recommended for all low-birthweight newborn babies.^1^ High-quality evidence has shown the benefits of these interventions on child survival, health, and development. The COVID-19 pandemic, caused by SARS-CoV-2 has, however, raised concern about the possibility and effect of SARS-CoV-2 transmission through close contact between mothers and their infants and breastfeeding. As of Aug 14, 2020, analyses of breastmilk samples of 175 mothers with confirmed SARS-CoV-2 infection have been reported,^2^ with SARS-CoV-2 RNA identified by RT-PCR in samples from ten mothers. However, evidence of infectious virus that is capable of replicating and infecting other cells^3^ and mother–infant transmission through breastmilk has not been shown.

Interpretation of existing evidence and how it should shape public health policy is challenging because the population effects and long-term health outcomes of COVID-19 among mothers and infants are uncertain. WHO interim guidance^4^ (May 27, 2020), on the basis of available evidence, recommends that “mothers with suspected or confirmed COVID-19 should be encouraged to initiate and continue breastfeeding”, while implementing infection control measures, and “should not be separated from their infants unless the mother is too sick to care for her baby”. The guidance notes that the severity of COVID-19 infections is much lower in infants than in adults and that “COVID-19 in infants and children represents a much lower risk to survival and health than the other infections and conditions that breastfeeding is protective against”. Some national health agencies, however, have advised separation of infants from mothers with suspected or confirmed SARS-CoV-2 and avoidance of breastfeeding^5^, ^6^—although some have revised their position. A Cochrane review of 19 national policies reported no consensus regarding whether breastfeeding should be contraindicated among mothers with confirmed or suspected COVID-19 and even among asymptomatic mothers with unknown COVID-19 status.^7^ Reports of SARS-CoV-2 RNA in breastmilk, even without evidence of transmission, have fuelled uncertainty and anxiety and even led some authors to recommend against breastfeeding.^8^ Unsurprisingly, health workers and communities are confused about appropriate infant feeding recommendations.^9^ In some settings, local policies to prevent COVID-19 have resulted in delays in initiation of and disruption in breastfeeding among mothers with unknown COVID-19 status.^9^ Furthermore, the pandemic and related evidence gaps and anxieties are egregiously being exploited as a marketing opportunity by the breastmilk substitute industry.^10^, ^11^

## An approach for deciding public health policy

Even in the absence of high-quality data, public health policy should, to the extent possible, be evidence-based. We present an approach based on available evidence for the competing benefits and harms (panel) for developing policy on mother–infant contact and infant feeding practices in the context of COVID-19, or for other viral agents that might appear in the future, that balances the risks associated with viral infection with the effect on child survival, lifelong health, and development. Considerations include the incidence among mothers, duration of infectivity, feasibility of identifying infection in a timely manner, SARS-CoV-2 transmission risks, and effects of infection in infants, alongside the mortality and health risks of separation and not breastfeeding. In time, relevant data will become available and should be interpreted while recognising complementary effects of these considerations and dependent outcomes. Here, we consider the risk of exposure to SARS-CoV-2 that is associated with close contact and breastfeeding and compare this risk with the risks of no contact between the mother and infant and avoidance or stopping of breastfeeding (and use of breastmilk substitute).

Panel **Available evidence and key research and programme gaps**

**SARS-CoV-2 infection fatality rate**

*Available evidence*

• Preliminary data from south Asia and Latin America indicate very low infection fatality rates in infants and young children (appendix p 7)

*Research and programme gaps*

• What is the infection fatality rate of severe acute respiratory syndrome coronavirus 2 (SARS-CoV-2) in infants and young children by age, nutritional status, comorbidity, socio-economic setting and health system capacity?

**Mortality and long-term health outcomes of mother–infant contact and breastfeeding**

*Available evidence*

• High quality data show survival and long-term health benefits that are associated with early initiation of and exclusive and continued breastfeeding (cumulative benefits with longer durations of breastfeeding) and with mother–infant contact (appendix p 7)
• Breastfeeding rates decrease when health-care workers communicate mixed messages (appendix p 7)
• Breastfeeding rates decrease as a result of marketing of breastmilk substitutes (appendix p 7)

*Research and programme gaps*

• What are the protective effects of specific anti-SARS-CoV-2 factors in breastmilk?

**SARS-CoV-2 transmission risk through mother–infant contact and breastfeeding**

*Available evidence*

• SARS-CoV-2 RNA identified intermittently in breastmilk
• Evidence for transmission competent virus not reported in breastmilk (appendix pp 7–8)
• No transmission through breastfeeding reported
• COVID-19 neonatal deaths mostly among preterm babies and when the mother is seriously unwell (and therefore separated; appendix pp 7–8)
• SARS-CoV-2 antibodies identified in breastmilk (appendix pp 7–8)
• Lactoferrin and many other anti-infectious molecules (eg, secretory leucocyte protease inhibitor and lysozymes) present in breastmilk with potential anti-SARS-CoV-2 activity (appendix pp 7–8)

*Research and programme gaps*

• Is active transmission-competent virus present in breastmilk?
• If present, how frequently, for how long, and what is the relationship to symptoms (before and after symptoms)?
• What is the transmission rate through respiratory droplets and close contact by infant age and time in contact with mother when asymptomatic or infectious?
• What is the additional transmission rate through breastfeeding by infant age and amount of breastmilk consumed?
• What is the ability of virus to overcome host defences according to infant age?

**Identification of mothers with confirmed SARS-CoV-2 infection**

*Available evidence*

• Testing done only once symptoms present or during a contact tracing process (eg, when a relative or other household member has symptoms or tests positive for infection; appendix p 8)
• Some centres doing tests in all mothers who present in labour (appendix p 8)
• Time to return of results 24–72 h or longer, although some accredited tests provide PCR result within 60 min (GenXpert and others; appendix p 8)
• Testing not available in many facilities, including high-resource settings (appendix p 8)
• 5–20% of individuals with suspected COVID-19 are likely to test positive for SARS-CoV-2;^12^ in other reports, 52–96% of individuals who were tested because of symptoms suggestive of COVID-19 were negative and had a different cause of symptoms (appendix p 8)

*Research and programme gaps*

• What is the transmission risk associated with mother–infant contact and other caregivers and household members while the mother is asymptomatic or with early symptoms (pre-results)?
• What are the best approaches for rapid testing?

### Policy considerations for mothers with confirmed SARS-CoV-2

On consideration of a balance of risk, public health policy would favour separation and avoiding breastfeeding among mothers with confirmed SARS-CoV-2 if evidence exists of substantial, immediate or potential long-term adverse health effects of COVID-19 in infants or young children; of substantive SARS-CoV-2 transmission through mother–infant contact or breastfeeding; and identification of mothers with SARS-CoV-2 while infectious, with or without symptoms. These conditions are all necessary if avoidance of contact and breastfeeding are to produce an overall benefit.

Conversely, public health policy for mothers with confirmed SARS-CoV-2 infection would favour continued breastfeeding and mother–infant contact if evidence exists for substantial risk of immediate infant or child death or long-term adverse effects associated with separation and non-breastfeeding for the infant or mother; few negative health effects of COVID-19 among infants or young children; and low risk of SARS-CoV-2 transmission through mother–infant contact and breastfeeding.

Mother–infant contact and breastfeeding are normative recommendations and therefore standard of care. If any of the inputs that justify separation and non-breastfeeding are absent, then the chain of effects supporting a policy that is different from standard care would be broken. For mothers with suspected COVID-19 but who have not been tested or confirmed, justification for a policy in favour of separation and avoiding breastfeeding would need evidence of a high likelihood of SARS-CoV-2 infection in those with suspected infection and even greater harm associated with COVID-19 to allow for false positives. A policy that is in favour of separation and non-breastfeeding should also address the potential for altered feeding practices in the general population—ie, diminished breastfeeding because of public health mixed messaging and confusion among health workers and beliefs based on risk aversion.

## COVID-19 chain of risks and effects of policy options: illustrative analyses

Here, we consider the effects of inputs that sequentially and cumulatively influence the balance of risks for infants living in low-income and middle-income countries (LMICs). We estimated the effects of separation and non-breastfeeding by mothers with confirmed SARS-CoV-2 (with or without symptoms), identified either through universal testing of mothers of newborn babies or through testing of mothers presenting with COVID-19 symptoms. We estimated the potential number of infant deaths in 119 LMICs (appendix pp 4–6) that annually might be attributable to COVID-19 as well as the additional deaths due to separation and non-breastfeeding among infants who might be affected by such a policy approach. We also present data that are specific for 14 countries that show regional differences in neonatal and child mortality. We assumed a SARS-CoV-2 incidence rate among mothers of 10%, although this figure is based on reported prevalence.^13^, ^14^ In the absence of reported incidence data, this figure therefore overestimates actual incidence rates. SARS-CoV-2 transmission rates through close contact between a mother who is infected but pre-symptomatic or persistently asymptomatic and her infant are unknown. A systematic review^15^ of 40 studies reported a mean overall household secondary attack rate of 18·8% (95% CI 15·4–22·2; median 16·3% [IQR 10·5–24·0]). Rates were higher from symptomatic than from asymptomatic index cases, and lower to child contacts than to adult contacts. The highest reported household secondary attack rate was 44·6%.^16^ SARS-CoV-2 RNA has been detected intermittently in breastmilk, but replication-competent virus that is capable of infecting other cells and presumably able to cause transmission has not been reported.^3^ For these estimates, we examined the effect of transmission probabilities of 20% and 30%. SARS-CoV-2 infection fatality rates (IFRs) among infants identified through universal screening of mothers are also unknown. We, therefore, used case fatality rates (CFRs) derived from infants and children who were symptomatic for COVID-19, tested, and received medical care. In a review of 7780 children in 26 countries with confirmed COVID-19, the CFR was 0·09%.^17^ Using CFR instead of IFR will therefore overestimate the number of deaths potentially attributable to SARS-CoV-2 infection.

We used the Lives Saved Tool (LiST) and current national mortality rates to estimate infant deaths in 2020–21 that were attributable to separation and non-breastfeeding among approximately 104 000 000 livebirths in 119 LMICs. LiST is a modelling tool for estimating direct and indirect effects of changes in coverage of health interventions, including skin-to-skin contact; exclusive, partial, and predominant breastfeeding in infants younger than 6 months; and any breastfeeding from age 6 months to 23 months on infant and child mortality (appendix pp 2–3).^18^ High-income countries were not included. Breastfeeding prevalence rates in LiST are based on country-specific data and are organised by four age groups: 0–<1 month, 1–5 months, 6–11 months, and 12–24 months. The effect sizes of breastfeeding on survival vary by time of initiation, age, and category (ie, exclusive, predominant, or partial).^19^, ^20^ We examined two scenarios among infants of mothers with SARS-CoV-2 infection that was identified immediately after birth—eg, in the context of universal screening of mothers and also infants of mothers identified through testing if symptomatic in the first 12 months after birth.

The first scenario was no initiation of breastfeeding among exposed newborn babies and older infants stopping breastfeeding, and no neonates or older infants subsequently restarting breastfeeding after the mother is no longer infectious—eg, if the mother received no counselling or other support to help restart breastfeeding. The second scenario involved early initiation of breastfeeding among all newborn babies; exposed newborn babies and older infants stopping breastfeeding; 50% of neonates and older infants restarting breastfeeding after mothers are no longer infectious (eg, the mother receives good counselling support and is able to restart breastfeeding); and considering the effects of non-breastfeeding projected through the first 12 months of life.

### Infant mortality attributable to COVID-19 or a policy of separation and non-breastfeeding among mothers with confirmed SARS-CoV-2 infection

Based on existing evidence and the assumptions already described, annual deaths potentially attributable to COVID-19 in infants younger than 12 months in LMICs would be approximately 1875 or 2809 globally depending on whether the transmission probability is assumed to be 20% or 30%. Estimated deaths in infants aged 0–12 months who might be affected by a policy of separation and non-breastfeeding among mothers with confirmed SARS-CoV-2 infection are presented in the table. Deaths among infants affected by a policy of separation and non-breastfeeding would be at least 67 times greater than mortality potentially attributable to COVID-19 (table). Assuming 30% transmission, one would need to avoid mother–infant contact and breastfeeding among 3700 infants to prevent one COVID-19-attributable death—ie, the number of newborn babies and infants who might be exposed to a policy requiring separation and non-breastfeeding in the event of maternal SARS-CoV-2 infection divided by the number of COVID-19 attributable deaths (table).

**Table:** Estimated additional infant deaths (2020–21)

	**Livebirths**	**Neonatal deaths (per 1000 livebirths), 2018–19**	COVID-19-related deaths in infants*‡‡	COVID-19-related deaths in infants*§§	Additional infant*deaths because of early separation and no breastfeeding among exposed newborns and infants	
					Scenario 1	Scenario 2
Ghana	864 720	23·95	16	23	1983	1311
Nigeria	7 197 225	36·02	130	194	49 518	35 117
South Africa	1 204 852	10·74	22	33	1300	827
Argentina	747 792	6·36	13	20	123	75
Guatemala	425 986	12·30	8	12	685	509
Mexico	2 229 502	7·51	40	60	615	367
Lebanon	116 540	4·32	2	3	16	11
Pakistan	5 798 236	41·95	104	157	18 403	12 208
Turkey	1 320 135	5·46	24	36	182	118
Indonesia	4 749 802	12·74	85	128	5809	4015
Papua New Guinea	234 195	22·11	4	6	666	461
Thailand	709 759	5·02	13	19	95	62
India	23 724 430	22·73	427	641	46 937	31 928
Myanmar	942 842	36·74	17	25	2777	1926
Sri Lanka	326 879	4·49	6	9	49	34
All LMICs (119 countries)	104 051 261	17·67	1875	2809	273 453	188 626

CFR=case fatality rate. LMIC=low-income and middle-income countries. Data are n.

†Assuming 20% transmission rate and a CFR of 0·09%.

* Age 0–12 months.

‡ Assuming no separation and continued breastfeeding.

§ Assuming 30% transmission rate and a CFR of 0·09%.

## Discussion

Even assuming high rates of mother–infant SARS-CoV-2 transmission through contact and breastmilk and using CFR instead of IFR, the additional deaths among newborn babies and infants in LMICs who would be subjected to separation and non-breastfeeding in either scenario (188 000 or 273 000) would be approximately 67 times greater than the number of newborn babies and infants who are likely to die because of COVID-19. Because we assume higher incidence, transmission, and mortality associated with COVID-19 among newborn babies and infants than has been reported to date, this estimate is almost certainly an underestimate of the effect on mortality of a policy that would separate mothers from their newborn babies and disrupt breastfeeding. A low, or even very low, CFR in SARS-CoV-2-infected infants and children is the primary influence on this estimate, although lower transmission risks among infants and children, as suggested by existing evidence, are also important. An observational study^21^ that included 116 mothers with confirmed SARS-CoV-2 reported no transmission among their newborn babies aged up to 1 month, despite rooming in, and 78% practising breastfeeding. Mothers also practised simple precautionary measures including wearing masks and hand hygiene.^21^ Although high-income countries are not included in LiST, the benefits of exclusive and continued breastfeeding and use of breastmilk—eg, for infants with necrotising enterocolitis and for reducing hospital admissions for infectious diseases—are also likely to greatly outweigh the risks of COVID-19 in infants and young children. The presence of SARS-CoV-2 RNA in breastmilk in the absence of other evidence indicating that the virus can be transmitted through breastmilk is an important observation, but public health decisions that are based on this finding alone are not justified.

The analyses only consider the immediate mortality consequences of the two public health approaches. Yet, the adverse effects of delays and interruptions of breastfeeding might extend beyond any period of transient cessation related to the risk for SARS-CoV-2 infection. Unless skilled breastfeeding counselling and support is available to individual mothers to help re-establish breastfeeding, temporary cessation is likely to diminish rates and duration of continued breastfeeding. Routine services have been disrupted by the pandemic in most settings and coverage of breastfeeding counselling is very likely to have also been affected.

Changes in feeding and care practice among mothers who are only suspected to have COVID-19, or by mothers of unknown status who are anxious or confused by the lack of accurate health messaging or through opportunistic marketing by the breastmilk substitute industry, will also indirectly increase mortality and losses in health status. If the non-survival benefits of breastfeeding for the child and mother^22^ are also considered, then much higher COVID-19 IFRs and transmission rates would be needed to justify a policy of separation and non-breastfeeding. Furthermore, separation of the infant from a mother with confirmed or suspected COVID-19 does not necessarily remove all transmission risks for the infant. Transmission might also occur from exposure to infected but asymptomatic carers at home, in health facilities, or in the community. In Brazilian national surveys, 13 (35%) of 37 family members of participants who tested positive for SARS-CoV-2 antibodies also tested positive,^23^ inferring that to effectively implement a policy of separation, infants would have to be removed from their homes, which is clearly unrealistic.

The absence of accurate risk data presents a challenge for making public health recommendations. Empirical data will, in time, inform where the thresholds between low and high risks fall. While CFRs among infants and children presenting to health facilities are becoming available, IFRs, in which asymptomatic individuals and cases of mild illness also contribute to the denominator, are unknown and are perhaps not being investigated in population surveys. Also, adverse outcomes in young infants appear to have occurred principally among neonates who were born preterm or whose mothers had moderate-to-severe symptoms of COVID-19, making it difficult to ascertain what is attributable to COVID-19 alone. In-utero transmission seems likely^24^ and might also bias postnatal outcome data, further complicating the interpretation of contact and infant feeding data in neonates. The protective effects of breastmilk against transmission of SARS-CoV-2 and the potential to mitigate infection through specific antibodies and anti-infectious agents in breastmilk are yet to be understood. However, components of breastmilk such as IgA, lactoferrin, and other anti-infectious molecules (eg, secretory leucocyte protease inhibitor and lysozymes) are likely to provide broad protection against viral invasion.

The COVID-19 pandemic shows how evidence with respect to infant feeding and care practices can be inconsistently assessed. The lack of consensus in recommendations and public health messaging suggests that risk aversion to the possible effects of COVID-19 have outweighed consideration of the survival and health benefits that skin-to-skin contact and breastfeeding offer to newborn babies and young infants. These mixed messages and concerns echo some of the perspectives and disagreement that occurred during the HIV epidemic when, in some settings, avoidance of postnatal transmission was prioritised at almost any cost. Yet the transmission risks and consequences of COVID-19 are very different from those of HIV.^25^ The COVID-19 pandemic shows how approaches to policy making, whether in the context of COVID-19 or other infectious diseases, need to consider the full balance of risks, including the consequences of interrupting evidence-based infant-feeding practices and other standards of care.

## Conclusions

On the basis of available evidence, including programmatic information and illustrative analyses, we make five conclusions. First, the state of evidence and balance of risk estimates support mother–infant close contact and breastfeeding by mothers with confirmed SARS-CoV-2 infection while still implementing infection prevention and control measures, including handwashing and wearing face masks.^4^ The survival benefits of breastfeeding substantially outweigh the very low reported CFRs among infants with COVID-19.^4^ Second, public health authorities should consider the full scope of evidence and implications for all-cause child mortality and other health outcomes, and ensure that resultant policy and associated messaging are coherently communicated to health-care workers and communities. Third, research will populate the evidence gaps and future estimations should adopt a comprehensive child survival and health framing to avoid oversimplification. Animal models would be helpful for elucidating infectivity of viruses through breastfeeding. Fourth, in the COVID-19 pandemic, breastfeeding counselling and support and other interventions and approaches should focus on how to reduce the small risk of transmission and effect through respiratory spread. Fifth, public health authorities and legislators should proactively address the deliberate exploitation and seeding of doubt about breastfeeding by commercial interests.

Future epidemics caused by novel viruses will probably involve similar challenges. The approach that we have presented will be useful for planning concerted advice that is globally consistent on risks and benefits associated with breastfeeding in light of potential viral transmission.
