# Supplementary material for: A public health approach for deciding policy on infant feeding and mother–infant contact in the context of COVID-19
Source: Lancet Glob Health. 2021 Feb 22;9(4):e552–7. doi: 10.1016/S2214-109X(20)30538-6 (PMC7906661; doi:10.1016/S2214-109X(20)30538-6)
Supplement: Supplementary appendix [file mmc1.pdf]

THE LANCET
Global Health

Supplementary appendix

This appendix formed part of the original submission and has been peer reviewed. We post it as supplied by the authors.

Supplement to: Rollins N, Minckas N, Jehan F, et al. A public health approach for deciding policy on infant feeding and mother–infant contact in the context of COVID-19. *Lancet Glob Health* 2021; published online Feb 22. http://dx.doi.org/10.1016/S2214-109X(20)30538-6.

Appendix: Modelling inputs and assumptions for LiST / References (17 February 2021)

LiST is a deterministic, linear, mathematical model that allows to estimate the impact of coverage change on mortality. LiST produces the same outputs each time the model is run with identical inputs.[1]

Calculation of the impact of coverage change on mortality used by LiST:
CAUSE-SPECIFIC MORTALITY x COVERAGE CHANGE x AFFECTED FRACTION x EFFECTIVENESS=
**LIVES SAVED/LIVES LOST**

Assumptions, inputs and sources:

Cause specific mortality:

Calculated as: Births x Mortality rate x Proportion of deaths due to specific cause

Sources:

Births: according to the latest national estimates (find table with country specific sources below)
Mortality rate: UN Inter-agency Group for Child Mortality Estimation (IGME). https://childmortality.org/
Cause of deaths: WHO Maternal and Child Epidemiology Estimations (MCEE).
https://www.who.int/healthinfo/global_burden_disease/estimates/en/index1.html

Coverage change:

Scenario 1: Assumptions:

- Early initiation of breastfeeding stays at national prevalence for general population.
- Exposed newborns (10%) do not initiate breastfeeding.
- All exposed (10%) newborns and infants stop breastfeeding at each age group (0-1 month, from 1-5 months, from 6-11 months) and do not restart.

Scenario 2: Assumptions:

- Early initiation of breastfeeding stays at national prevalence.
- All exposed newborns and infants (10%) stop breastfeeding at each age group (0-1 month, from 1-5 months, from 6-11 months). Note. No mortality attributed to temporary disruption of breastfeeding in the 50% of exposed newborns and infants who restart breastfeeding.
- 50% of exposed newborns and infants do not restart, carrying the effect of the disruption to the next period (in exposed populations by age group: 50% reduction from <1 month, 75% reduction from 1-5 months [includes reduced BF rate from <1m], 75% reduction from 6-11 months [includes reduced BF rate from <1m]).

Affected fraction[2]:

Assumed at 100% of exposed newborns and infants as survival of all infants 0-1 months, 1-55 months, 6-11 months can be affected by changes in breastfeeding coverage.

---

[1] Walker, N., Tam, Y. & Friberg, I.K. Overview of the Lives Saved Tool (LiST). BMC Public Health 13, S1 (2013). https://doi.org/10.1186/1471-2458-13-S3-S1

[2] Proportion of cause specific-deaths that can be averted by a specific intervention.

Effectiveness:

Table S1. Effect size of breastfeeding on mortality by age group and type of feeding

		Predominant	*Partial*	*No breastfeeding*
Early initiation*	*1 (ref)*	*1*	*1*	*5.4 (2.3;13.0)*
Late initiation*	*1.35 (1.13,1.62)*	*1.35 (1.13,1.62)*	*1.35 (1.13,1.62)*	*5.4 (2.3;13.0)*
0-5 months**	*1 (ref)*	*1.48 (1.14;1.92)*	*2.84 (1.63, 4.97)*	*14.4 (6.13, 33.86)*
6-23 months**	*1 (ref)*			*3.69 (1.49, 9.17)*

**risk estimated for infectious causes of mortality in neonatal period (diarrhea, sepsis and pneumonia)*

*** risk estimated for all-cause mortality*

Table S2. Impact of sub-optimal breastfeeding on cause-specific mortality in neonates

	Early initiation of breastfeeding Relative risk	Late initiation of breastfeeding Relative risk
**Neonatal - Diarrhea**		
Exclusive breastfeeding	1	1.35
Predominant breastfeeding	1	1.35
Partial breastfeeding	1	1.35
Not breastfeeding	5.4	5.4
**Neonatal - Sepsis**		
Exclusive breastfeeding	1	1.35
Predominant breastfeeding	1	1.35
Partial breastfeeding	1	1.35
Not breastfeeding	5.4	5.4
**Neonatal - Pneumonia**		
Exclusive breastfeeding	1	1.35
Predominant breastfeeding	1	1.35
Partial breastfeeding	1	1.35
Not breastfeeding	5.4	5.4

Table S3. Impact of sub-optimal breastfeeding on cause-specific mortality in children 1-59 months

	1-5 months Relative risk	6-11 months Relative risk	12-23 months Relative risk	24-59 months Relative risk
**Diarrhea**				
Exclusive breastfeeding	1	1	1	1
Predominant breastfeeding	2.28	1	1	1
Partial breastfeeding	4.62	1	1	1
Not breastfeeding	10.52	1.47	2.57	1
**Pneumonia**				
Exclusive breastfeeding	1	1	1	1
Predominant breastfeeding	1.66	1	1	1
Partial breastfeeding	2.5	1	1	1
Not breastfeeding	14.97	1.92	1.92	1
**Meningitis**				
Exclusive breastfeeding	1	1	1	1
Predominant breastfeeding	1.48	1	1	1
Partial breastfeeding	2.84	1	1	1
Not breastfeeding	14.4	3.69	3.69	1
**Measles**				
Exclusive breastfeeding	1	1	1	1
Predominant breastfeeding	1.48	1	1	1
Partial breastfeeding	2.84	1	1	1
Not breastfeeding	14.4	3.69	3.69	1
**Pertussis**				
Exclusive breastfeeding	1	1	1	1
Predominant breastfeeding	1.48	1	1	1
Partial breastfeeding	2.84	1	1	1
Not breastfeeding	14.4	3.69	3.69	1

Sources:

Lamberti LM, Zakarija-Grković I, Fischer Walker CL, Theodoratou E, Nair H, Campbell H, Black RE. Breastfeeding for reducing the risk of pneumonia morbidity and mortality in children under two: a systematic literature review and meta-analysis. BMC Public Health. 2013;13 Suppl 3(Suppl 3):S18. doi: 10.1186/1471-2458-13-S3-S18. Epub 2013 Sep 17.

Black RE, Victora CG, Walker SP, Bhutta ZA, Christian P, de Onis M, Ezzati M, Grantham-McGregor S, Katz J, Martorell R, Uauy R; Maternal and Child Nutrition Study Group. Maternal and child undernutrition and overweight in low-income and middle-income countries. Lancet. 2013 Aug 3;382(9890):427-451. doi: 10.1016/S0140-6736(13)60937-X. Epub 2013 Jun 6. Erratum in: Lancet. 2013. 2013 Aug 3;382(9890):396.

Table S4. Country estimates used by LiST

Country	Data source
Afghanistan	DHS 2015
Albania	DHS 2017-2018
Algeria	MICS 2012
Angola	DHS 2015
Argentina	MICS 2011
Armenia	DHS 2015-2016
Azerbaijan	DHS 2016
Bangladesh	MICS 2019
Belarus	MICS 2012
Belize	MICS 2015
Benin	DHS 2017-2018
Bhutan	MICS 2010
Bolivia	DHS 2008
Bosnia and Herzegovina	MICS 2011
Botswana	Family Health Survey 2007
Brazil	PHDS 2006
Burkina Faso	DHS 2010
Burundi	DHS 2016
Cape Verde	DHS 2005
Cambodia	DHS 2014
Cameroon	MICS 2014
Central African Republic	MICS 2010
Chad	DHS 2014
Colombia	DHS 2015
Comoros	DHS 2012
Congo	MICS 2014
Costa Rica	MICS 2011
Côte d'Ivoire	MICS 2016
Cuba	MICS 2014
Djibouti	MICS 2006
Dominican Republic	MICS 2014
Democratic Republic of the Congo	DHS 2013
Ecuador	NSS 2012
Egypt	DHS 2014
El Salvador	MICS 2014
Equatorial Guinea	DHS 2011
Eritrea	DHS 2002
Ethiopia	DHS 2016
Gabon	DHS 2012
Gambia	MICS 2018

Georgia	MICS 2018
Ghana	DHS 2014
Guatemala	DHS 2014
Guinea	DHS 2018
Guinea-Bissau	MICS 2014
Guyana	MICS 2014
Haiti	DHS 2016
Honduras	DHS 2011
India	DHS 2015
Indonesia	DHS 2012
Iraq	MICS 2018
Jamaica	MICS 2011
Jordan	PFHS 2017-2018
Kazakhstan	MICS 2015
Kenya	DHS 2014
Kiribati	DHS 2009
Dem. People's Republic of Korea	MICS 2017
Kyrgyzstan	MICS 2018
Lao People's Democratic Republic	MICS 2017
Lebanon	No household data*
Lesotho	MICS 2018
Liberia	DHS 2013
Madagascar	MDG Survey 2012
Malawi	DHS 2015
Maldives	DHS 2016-2017
Mali	MICS 2015
Mauritania	MICS 2015
Mexico	MICS 2015
Republic of Moldova	MICS 2012
Mongolia	MICS 2018
Montenegro	MICS 2018
Morocco	DHS 2003
Mozambique	DHS 2015
Myanmar	DHS 2015
Namibia	DHS 2013
Nepal	DHS 2016
Nicaragua	RHS 2006
Niger	DHS 2012
Nigeria	DHS 2018
Republic of North Macedonia	MICS 2011
Pakistan	DHS 2017-2018
Panama	MICS 2013
Papua New Guinea	DHS 2016-2018

Paraguay	MICS 2016
Peru	DHS 2016
Philippines	DHS 2017
Rwanda	DHS 2014
Saint Lucia	MICS 2012
Samoa	DHS 2009
São Tomé and Príncipe	MICS 2014
Senegal	DHS 2017
Serbia	MICS 2014
Sierra Leone	MICS 2017
Solomon Islands	DHS 2006
Somalia	MICS 2006
South Africa	DHS 2016
South Sudan	MICS 2010
Sri Lanka	DHS 2016
Sudan	MICS 2014
Suriname	MICS 2010
Swaziland	MICS 2014
Syrian Arab Republic	MICS 2006
Tajikistan	DHS 2017-2018
United Republic of Tanzania	DHS 2015
Thailand	MICS 2015
Timor-Leste	DHS 2016
Togo	DHS 2013
Tonga	DHS 2012
Tunisia	MICS 2018
Turkey	DHS 2013
Turkmenistan	MICS 2015
Uganda	DHS 2016
Ukraine	MICS 2012
Uzbekistan	MICS 2006
Vanuatu	DHS 2013
Viet Nam	MICS 2013-2014
Yemen	DHS 2013
Zambia	DHS 2018
Zimbabwe	MICS 2019

*regional estimates were used

References for panel

SARS-CoV-2 infection fatality rate

Available evidence

• Preliminary data from south Asia and Latin America indicates very low infection fatality rates in infants and young children

<u>Sources</u>

- O'Driscoll M, Ribeiro Dos Santos G, Wang L, Cummings DAT, Azman AS, Paireau J, et al. Age-specific mortality and immunity patterns of SARS-CoV-2. Nature. 2021;590(7844):140-5
- Personal communication – Cesar Victora, Pelotas, Brazil (manuscript in preparation)

Mortality and long-term health outcomes of mother–infant contact and breastfeeding

Available evidence

• High quality data show survival and long-term health benefits that are associated with early initiation of and exclusive and continued breastfeeding (cumulative benefits with longer durations of breastfeeding) and with mother– infant contact
• Breastfeeding rates decrease when health-care workers communicate mixed messages
• Breastfeeding rates decrease as a result of marketing of breastmilk substitutes

<u>Sources</u>

- Khan J, Vesel L, Bahl R, Martines JC. Timing of breastfeeding initiation and exclusivity of breastfeeding during the first month of life: effects on neonatal mortality and morbidity--a systematic review and meta-analysis. Maternal and child health journal. 2015;19(3):468-79.
- Piwoz EG, Huffman SL. The Impact of Marketing of Breast-Milk Substitutes on WHO-Recommended Breastfeeding Practices. Food and nutrition bulletin. 2015;36(4):373-86
- Victora CG, Bahl R, Barros AJ, Franca GV, Horton S, Krasevec J, et al. Breastfeeding in the 21st century: epidemiology, mechanisms, and lifelong effect. Lancet. 2016;387(10017):475-90

SARS-CoV-2 transmission risk through mother–infant contact and breastfeeding

Available evidence

• SARS-CoV-2 RNA identified intermittently in breastmilk
• Evidence for transmission competent virus not reported in breastmilk
• No transmission through breastfeeding reported
• COVID-19 neonatal deaths mostly among preterm babies and when the mother is seriously unwell (and therefore separated)
• SARS-CoV-2 antibodies identified in breastmilk
• Lactoferrin and many other anti-infectious molecules (eg, SLPI and lysozymes) present in breastmilk with potential anti-SARS-CoV-2 activity

<u>Sources</u>

- Chambers C, Krogstad P, Bertrand K, Contreras D, Tobin NH, Bode L, et al. Evaluation for SARS-CoV-2 in Breast Milk From 18 Infected Women. JAMA. 2020;324(13):1347-8.
- Gross R, Conzelmann C, Muller JA, Stenger S, Steinhart K, Kirchhoff F, et al. Detection of SARS-CoV-2 in human breastmilk. Lancet. 2020;395(10239):1757-8.
- Fox A, Marino J, Amanat F, Krammer F, Hahn-Holbrook J, Zolla-Pazner S, et al. Robust and Specific Secretory IgA Against SARS-CoV-2 Detected in Human Milk. iScience. 2020;23(11):101735.
- Peng S, Zhu H, Yang L, et al. A study of breastfeeding practices, SARS-CoV-2 and its antibodies in the breast milk of mothers confirmed with COVID-19. The Lancet Regional Health - Western Pacific. 2020;4:100045. Published 2020 Nov 1. doi:10.1016/j.lanwpc.2020.100045
- Peroni DG, Fanos V. Lactoferrin is an important factor when breastfeeding and COVID-19 are considered. Acta paediatrica (Oslo, Norway : 1992). 2020;109(10):2139-40
- Picaud JC, Buffin R, Rigourd V, Boscher C, Lamireau D, Dumoulin D, et al. It's time to change the recommendations on COVID-19 and human milk donations. Acta paediatrica (Oslo, Norway : 1992). 2021. Feb 2. doi: 10.1111/apa.15782

- Salvatore CM, Han JY, Acker KP, Tiwari P, Jin J, Brandler M, et al. Neonatal management and outcomes during the COVID-19 pandemic: an observation cohort study. Lancet Child Adolesc Health. 2020;4(10):721-7

Identification of mothers with confirmed SARS-CoV-2 infection

Available evidence

- Testing done only once symptoms present or during a contact tracing process (eg, relative)
- Some centres doing tests in all mothers who present in labour
- Time to return of results 24–72 h or longer, although some accredited tests provide PCR result within 60 min (GenXpert and others)
- Testing not available in many facilities, including high-resource settings
- 5–20% of individuals with suspected COVID-19 are likely to test positive for SARS-CoV-2; in other reports, 52–96% of individuals who were tested because of symptoms suggestive of COVID-19 were negative and had a different cause of symptoms

Sources

- Abeysuriya S, Wasif S, Counihan C, Shah N, Iliodromiti S, Cutino-Moguel MT, et al. Universal screening for SARS-CoV-2 in pregnant women at term admitted to an East London maternity unit. European journal of obstetrics, gynecology, and reproductive biology. 2020;252:444-6.
- Buonafine CP, Paiatto BNM, Leal FB, de Matos SF, de Morais CO, Guerra GG, et al. High prevalence of SARS-CoV-2 infection among symptomatic healthcare workers in a large university tertiary hospital in Sao Paulo, Brazil. BMC Infect Dis. 2020;20(1):917.
- Cruz-Lemini, M.; Ferriols ; Perez, E.; de la Cruz Conty, M.L.; Caño Aguilar, A.; Encinas Pardilla, M.B.; Prats Rodríguez, P.; Muner Hernando, M.; Forcen Acebal, L.; Pintado Recarte, P.; Medina Mallen, M.d.C.; et al. Obstetric Outcomes of SARS-CoV-2 Infection in Asymptomatic PregnantWomen. Viruses 2021, 13, 112. https://doi.org/10.3390/v13010112
- Fairlie L, Sawry S, Patel F, Balkus JE, Kalk E, Mutevedzi P, et al. COVID-19 in pregnancy in South Africa: Tracking the epidemic and defining the natural history. South African medical journal = Suid-Afrikaanse tydskrif vir geneeskunde. 2020;110(8):729-31
- Ferrazzi E, Beretta P, Bianchi S, Cetin I, Guarnerio P, Locatelli A, et al. SARS-CoV-2 infection testing at delivery: a clinical and epidemiological priority. The journal of maternal-fetal & neonatal medicine : the official journal of the European Association of Perinatal Medicine, the Federation of Asia and Oceania Perinatal Societies, the International Society of Perinatal Obstet. 2020:1-3
- Lan FY, Filler R, Mathew S, Buley J, Iliaki E, Bruno-Murtha LA, et al. COVID-19 symptoms predictive of healthcare workers' SARS-CoV-2 PCR results. PLoS One. 2020;15(6):e0235460
- Menni C, Valdes AM, Freidin MB, Sudre CH, Nguyen LH, Drew DA, et al. Real-time tracking of self-reported symptoms to predict potential COVID-19. Nat Med. 2020;26(7):1037-40
- Ondoa P, Kebede Y, Loembe MM, Bhiman JN, Tessema SK, Sow A, et al. COVID-19 testing in Africa: lessons learnt. Lancet Microbe. 2020;1(3):e103-e4.
- Pollan M, Perez-Gomez B, Pastor-Barriuso R, Oteo J, Hernan MA, Perez-Olmeda M, et al. Prevalence of SARS-CoV-2 in Spain (ENE-COVID): a nationwide, population-based seroepidemiological study. Lancet. 2020;396(10250):535-44
- Prabhu M, Cagino K, Matthews KC, Friedlander RL, Glynn SM, Kubiak JM, et al. Pregnancy and postpartum outcomes in a universally tested population for SARS-CoV-2 in New York City: a prospective cohort study. BJOG. 2020;127(12):1548-56.
- Reusken CB, Buiting A, Bleeker-Rovers C, Diederen B, Hooiveld M, Friesema I, et al. Rapid assessment of regional SARS-CoV-2 community transmission through a convenience sample of healthcare workers, the Netherlands, March 2020. Euro Surveill. 2020;25(12)1. Prabhu M, Cagino K, Matthews KC, Friedlander RL, Glynn SM, Kubiak JM, et al. Pregnancy and postpartum outcomes in a universally tested population for SARS-CoV-2 in New York City: a prospective cohort study. BJOG. 2020;127(12):1548-56
- Sutton D, Fuchs K, D'Alton M, Goffman D. Universal Screening for SARS-CoV-2 in Women Admitted for Delivery. N Engl J Med. 2020;382(22):2163-4.
- Wise J. Covid-19: What's going wrong with testing in the UK? BMJ 2020;370:m3678 doi: https://doi.org/10.1136/bmj.m3678 (Published 21 September 2020) [Last accessed 12 February 2021])
